# Analysis of Equation of States for the Suitability at High Pressure: MgO as an Example

**DOI:** 10.1155/2014/289353

**Published:** 2014-01-16

**Authors:** Kuldeep Kholiya, Jeewan Chandra, Swati Verma

**Affiliations:** ^1^Department of Applied Science, B.T. Kumaon Institute of Technology, Dwarahat 263653, India; ^2^Department of Applied Science, G.B. Pant Engineering College Ghurdauri, Pauri Garhwal 246194, India; ^3^Department of Computer Science, B.T. Kumaon Institute of Technology, Dwarahat 263653, India

## Abstract

A simple theoretical model is developed to study the high pressure behavior of solids and is applied to evaluate the pressure for MgO in case of large compression along with Shanker, Tait, Vinet, and Birch-Murnaghan equation of states (EOSs). These EOSs are also tested for the basic requirements revealed from the fundamental thermodynamics for solids in the limit of extreme compressions, as given by Stacey. It is found that for the high pressure compression behavior of MgO the present model, Tait, Vinet, and Birch-Murnaghan EOSs give the results compatible with the experimental data. It has also been found that in the regime of ultrahigh pressure the present model and Birch-Murnaghan EOS satisfy the Stacey criterion.

## 1. Introduction

Strength and elastic properties of a solid depend on the strength of its interatomic forces. Therefore, the application of pressure which changes interatomic distance of the substances changes its physical properties. This pressure versus volume relation is known as equation of state and may be quite useful to understand the physical properties of the material. In the literature, there are a number of equations of state, and these arise from an unchecked and unverifiable assumption concerning an assumed interatomic potential, an assumed strain function, or an assumed boundary condition that cannot be tested [1]. Therefore, the fitting of the experimental pressure-volume data has been the usual practice to prove the suitability of an EOS in different class of solids. However, the fitting of *P*-*V* data up to the small pressure range does not provide any support for the validity of the EOS beyond the fitting range due to two reasons.

- For small compression almost every EOS gives the results within the experimental uncertainty, as laboratory measured *P*-*V* data are often subjected to pressure calibration errors [2].
- Almost every EOS, new or old, can be fitted to a given range of *P*-*V* data by adjusting the values of the zero pressure, isothermal bulk modulus (*B*_0_), and its pressure derivatives (*B*_0_′, *B*_0_′′) [2].

Thus, the aim of the present work is to discuss the applicability of Shanker [3], Tait [4, 5], Vinet [6], and Birch-Murnaghan EOSs [7] along with an equation of state recently formulated by Kholiya [8] as well as to check the validity of these EOSs for Stacey criterion under infinite pressure. For this purpose MgO is taken as an example due to the reasons that the phase transition pressure for it is greater than 300 GPa [9, 10] and a large number of experimental and theoretical studies [9–33] have done for it so a large number of data is available up to the very large pressure for the sake of comparison.

## 2. Method of Analysis

On the basis of the findings that the repulsive branch of binding energy curves can be represented by a simple function of density Parsafar and Mason [34] have considered an EOS as

(1) $$\begin{array}{c} P{\left(\frac{V}{V_{0}}\right)}^{2}=A_{0}+A_{1}{\left(\frac{V}{V_{0}}\right)}^{-1}+A_{2}{\left(\frac{V}{V_{0}}\right)}^{-2}, \end{array}$$

where *V*/*V*_0_ = *ρ*_0_/*ρ*, and *V*_0_, *ρ*_0_ are the zero pressure values of volume *V* and density *ρ*, respectively, and *A*_0_, *A*_1_, and *A*_2_ are constants at a particular temperature. On the basis of the first-principle calculations, using the augmented-plane-wave (APW) method and quantum statistical model Hama and Suito [35] revealed that the Parsafar-Mason EOS becomes less successful at high compressions (*V*/*V*_0_ < 0.65). In addition to this, Shanker and Kushwah [36] pointed out that the Parsafar and Mason EOS gives *P* as a fourth degree expression in *V*/*V*_0_; therefore, the determination of the higher-derivative properties such as the bulk modulus and its pressure derivatives becomes less convenient. Shanker and Kushwah [36] have expanded *PV* ^2^ in powers of [1 − (*V*/*V*_0_)] up to the quadratic term and found that the equation works well for materials having pressure derivative of the isothermal bulk modulus less than four. Recently, Kholiya [8] has expanded pressure in powers of density up to the quadratic term and achieved the EOS as

(2) P(V,T0) =B02[(B0′−3)−2(B0′−2)(VV0)−1+(B0′−1)(VV0)−2].

In the present study we have also considered Shanker, Tait, Vinet, and Birch-Murnaghan equation of states for the comparison purpose. These EOS may be given as [3–7]:

(3) $$\begin{array}{c} P\left(V,T_{0}\right)=B_{0}\left[\left(1-\frac{V}{V_{0}}\right)+\left(\frac{B_{0}^{\prime }+1}{2}\right){\left(1-\frac{V}{V_{0}}\right)}^{2}\right], \end{array}$$

(4) $$\begin{array}{c} P\left(V,T_{0}\right)=\frac{B_{0}}{B_{0}^{\prime }+1}\left[\exp\left\{\left(B_{0}^{\prime }+1\right)\left(1-\frac{V}{V_{0}}\right)\right\}-1\right], \end{array}$$

(5) $$\begin{array}{c} P=3B_{0}{\left(\frac{V}{V_{0}}\right)}^{-2/3}\left[1-{\left(\frac{V}{V_{0}}\right)}^{1/3}\right]\exp\left[X\left\{1-{\left(\frac{V}{V_{0}}\right)}^{1/3}\right\}\right], \end{array}$$

(6) P=32B0[(V0V)7/3−(V0V)5/3]×[1+34(B0′−4){(V0V)2/3−1}].

Equations (3), (4), (5), and (6) are Shanker, Tait, Vinet and Birch-Murnaghan equation of states, respectively, and here *B*_0_ and *B*_0_′ are the bulk modulus and its first order pressure derivative at *P* = 0 and *T* = *T*_0_, respectively, and *X* = (3/2)(*B*_0_′ − 1).

The bulk modulus may be given as, *B* = −*V*(*dP*/*dV*) so the expression for bulk modulus from (2)–(6) comes out as

(7) B=B0(B0′−2)(VV0)−1[(B0′−1B0′−2)(VV0)−1−1],B=B0(VV0)[1+(B0′+1){1−VV0}],B=B0(VV0)[exp{(B0′+1)(1−VV0)}],B=B0(VV0)−2/3[1+{X(VV0)1/3+1}{1−(VV0)1/3}]×exp[X{1−(VV0)1/3}],B=12B0[7(V0V)7/3−5(V0V)5/3]+38B0(B0′−4)[9(V0V)3−14(V0V)7/3+5(V0V)5/3].

The corresponding expressions for the first order pressure derivative of bulk modulus *B*′ = *dB*/*dP* obtained from (7) comes out as

(8) $$\begin{array}{c} B^{\prime }=\frac{\left(B_{0}^{\prime }-2\right)\left(V/V_{0}\right)-2\left(B_{0}^{\prime }-1\right)}{\left(B_{0}^{\prime }-2\right)\left(V/V_{0}\right)-\left(B_{0}^{\prime }-1\right)}, \end{array}$$

(9) $$\begin{array}{c} B^{\prime }=\frac{\left(B_{0}^{\prime }+1\right)\left(2V/V_{0}-1\right)-1}{\left(B_{0}^{\prime }+1\right)\left(1-\left(V/V_{0}\right)\right)+1}, \end{array}$$

(10) $$\begin{array}{c} B^{\prime }=\left(\frac{V}{V_{0}}\right)\left(B_{0}^{\prime }+1\right)-1, \end{array}$$

(11) B′=13[(V/V0)1/3(1−X)+2X(V/V0)2/31+{X(V/V0)1/3+1}{1−(V/V0)1/3} +X(VV0)1/3+2],

(12) B′=B08B[(B0′−4){81(V0V)3−98(V0V)7/3+25(V0V)5/3}  +43{49(V0V)7/3−25(V0V)5/3}].

All these EOSs are also tested for the basic criteria which must be satisfied by an EOS for its validity and applicability as suggested by Stacey [2, 37, 38]. These criteria are as follows.

- In the limit →*∞*, *V*/*V*_0_ → 0.
- With the increase in pressure isothermal bulk modulus must increase continuously and in the limit of infinite pressure *B* → *∞*.
- *B*′ must decrease progressively with the increase in pressure such that *B*′ remains greater than 5/3 in the limit of infinite pressure.

## 3. Results and Discussions 

To study the compression behavior the present study requires two input parameters, namely, *B*_0_, *B*_0_′. In the literature a lot of experimental values of *B*_0_ and *B*_0_′ are available for MgO for example, *B*_0_ = 160 [13], 161 [14], 160.2 [15], 161.4 [28], 160.2 [29], 153 [30], 160.2 [31], 161.3 [32] and *B*_0_′ = 4.15 [13], 3.94 [14], 3.99 [15], 4.29 [28], 4.15 [29], 4.1 [30], 4.03 [31], 4.24 [32]. In the low compression range by choosing the suitable set of input parameters all the equations can be fitted to give the experimental results as at low pressure all EOSs give almost the same results. But as pressure increases the difference of the values calculated from different EOSs increase. For high compression the small difference in *B*_0_ and *B*_0_′ has little effect on the calculated values and at high compression the form of an EOS becomes more significant and different EOSs give different results.

**Figure 1:**
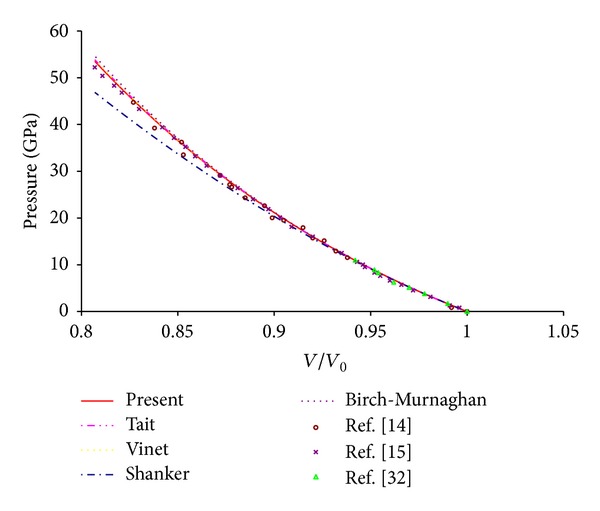
Compression behavior of MgO using different EOSs along with the experimental results.

**Figure 2:**
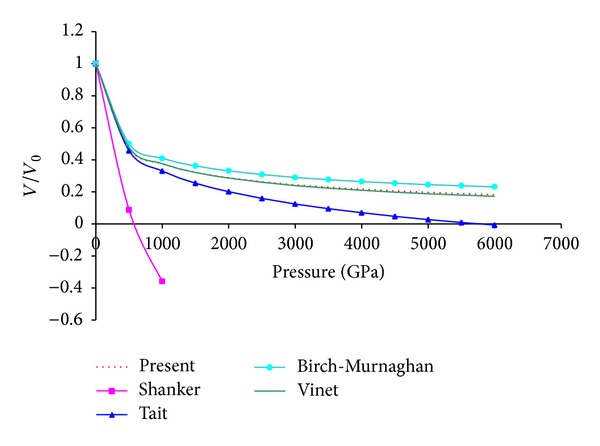
Compression behavior at ultrahigh pressure using different EOSs.

**Figure 3:**
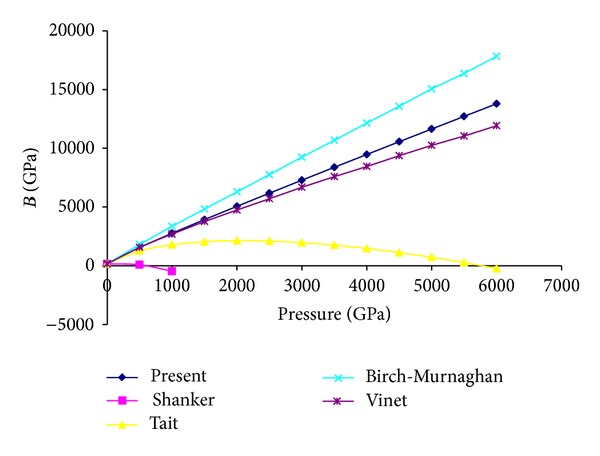
High pressure behavior of bulk modulus using different EOSs.

**Figure 4:**
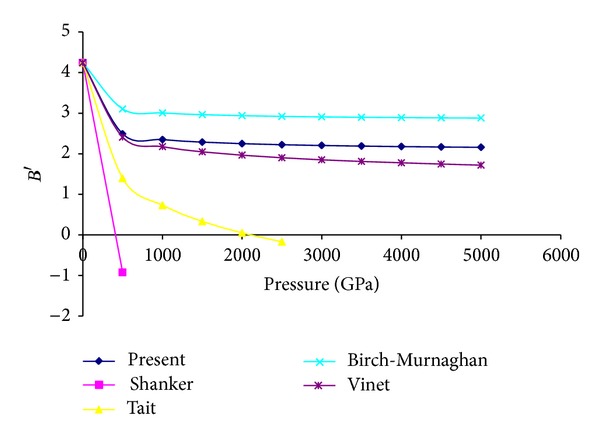
High pressure behavior of first order pressure derivative of bulk modulus using different EOSs.

In the present study the values of *B*_0_ and *B*_0_′ are taken to be 161.3 GPa and 4.24 as obtained by Li et al. [32], who measured the bulk modulus and its first order pressure derivative by fitting the experimentally measured velocity and density data to the third-order finite strain equations (Birch-Murnaghan EOS). The reason for this selection is that these values are the more recent values. The values of pressure calculated from (2) (present model), (3) (Shanker EOS), (4) (Tait EOS), (5) (Vinet EOS), and (6) (Birch-Murnaghan EOS) at different compression are plotted in Figure 1 along with the experimental findings. From Figure 1 it is clear that (2) (present model), (4) (Tait EOS), (5) (Vinet EOS), and (6) (Birch-Murnaghan EOS) give results compatible with the experimental findings while (3) (Shanker EOS) gives low results and hence fails at high pressure. It is pertinent to mention here that the selection of other set of input parameters also provides analogous outcome. It is important to mention here that for all the form of the EOSs value of *B*_0_ and *B*_0_′ is the same (161.3 GPa and 4.24) and the experimental values are taken from the respective papers. These EOS are also tested for the Stacey criteria arise from basic thermodynamic conditions.  Figure 2 shows the variation of *V*/*V*_0_ with pressure and from this figure it is clear that for Shanker and Tait EOSs *V*/*V*_0_ become zero at a finite value of pressure while for present, Vinet and Birch-Murnaghan EOS *V*/*V*_0_ becomes zero only in the limit of infinite pressure. At ultrahigh pressure it may also be noted that for compression the present model and Vinet EOS give almost similar results while Birch-Murnaghan EOS gives slightly higher results in comparison to these two EOSs. Here, it may be argued in favor of Shanker and Tait EOS that if any conventional solid were to approach infinite compression it would undergo dramatic phase transitions to exotic forms and equations of state do not carry through phase transitions but an equation of state must satisfy basic physical laws, in particular thermodynamic relationships; even outside the pressure ranges over which the materials that it describes can exist in the same phase because phase transition only changes the values of the parameters and not the basic thermodynamic relationship. The variation of isothermal bulk modulus *B* with pressure for MgO by using present, Shanker, Tait, Vinet, and Birch-Murnaghan EOSs is plotted in Figure 3. From Figure 3 it can be revealed that for Shanker and Tait EOSs initially *B* increases then decreases and finally becomes negative with the increase in pressure while for present Vinet and Birch-Murnaghan EOSs with the increase in pressure isothermal bulk modulus increases continuously and sharply so that it approaches to infinity in the limit of infinite pressure. At higher compression this continuous and sharp increase in bulk modulus may be attributed to the fact that at larger compressions the repulsive forces become so large.  Figure 4 represents the variation of first order pressure derivative of bulk modulus *B*′ with pressure. Again from this figure it is clear that for Shanker and Tait EOSs at high pressure *B*′ becomes negative while for present Vinet and Birch-Murnaghan EOSs it decreases progressively with the increase in pressure and then becomes asymptotic. From (8), (11), and (12) it may be noted that for present Vinet and Birch-Murnaghan EOSs the value of *B*_*∞*_′ (first order pressure derivative of bulk modulus at infinite pressure) comes out to be 2, 2/3, and 3, respectively. Therefore, Vinet does not follow the third criteria of the Stacey's basic criterion of an EOS that is, in the limit of infinite pressure *B*′ should remain greater than 5/3 while the present model and Birch-Murnaghan EOS follow all the three Stacey's criteria of an EOS.

## 4. Conclusions

From the overall discussion it may be concluded that the present formulation which was developed for nanomaterials [8] by expanding pressure in powers of density up to the quadratic term is also valid for the bulk materials. The formulation not only reproduces the experimental results regarding compression of bulk MgO but also satisfies the criteria based on the basic thermodynamic relations. Tait, Vinet, and Birch-Murnaghan EOSs give the results compatible with the experimental findings but Tait and Vinet EOSs fail to satisfy the basic criteria of an EOS. While at higher compression Shanker EOS does not give the satisfactory results and also fails when checked for the basic criteria of an EOS. Although, the Birch-Murnaghan EOS follows all the three criteria of an EOS, its shortcomings under very high pressure have been previously pointed out by Stacey [39]. Hence, the present formulation may be quite useful for studying the high pressure elastic behavior of solids.
